# Tripling the Bioavailability of Rosuvastatin Calcium Through Development and Optimization of an In-Situ Forming Nanovesicular System

**DOI:** 10.3390/pharmaceutics11060275

**Published:** 2019-06-11

**Authors:** Ibrahim Elsayed, Rania Moataz El-Dahmy, Ahmed Hassen Elshafeey, Nabaweya Abdelaziz Abd El Gawad, Omaima Naim El Gazayerly

**Affiliations:** 1 Department of Pharmaceutics and Industrial Pharmacy, Faculty of Pharmacy, Cairo University, Cairo 11562, Egypt; ibrahim.elsayed@pharma.cu.edu.eg (I.E.); ahmed.elshafeey@pharma.cu.edu.eg (A.H.E.); nabaweya.moussa@hotmail.com (N.A.A.E.G.); 2 Department of Pharmaceutical Sciences, College of Pharmacy, Gulf Medical University, Ajman 4184, UAE; 3 Department of Pharmaceutics, Faculty of Pharmacy, October 6 University, Cairo 12585, Egypt; dr.rania.moataz@hotmail.com

**Keywords:** in situ, Rosuvastatin calcium, bioavailability enhancement, mannitol and Aerosil

## Abstract

In situ forming nanovesicular systems (IFNs) were prepared and optimized to improve Rosuvastatin calcium (RC) oral bioavailability through increasing its solubility and dissolution rate. The IFN was composed of Tween^®^ 80 (T80), cetyl alcohol (CA), in addition to mannitol or Aerosil 200. A single simple step was adopted for preparation, then the prepared formulations were investigated by analyzing their particle size (PS), polydispersity index (PDI), Zeta potential (ZP), entrapment efficiency (EE), and flowability properties. D-optimal design was applied to choose the optimized formulations. The maximum desirability values were 0.754 and 0.478 for the optimized formulations containing 0.05 g CA, 0.18 g T80, and 0.5 g mannitol (OFM) or Aerosil (OFA), respectively. In vitro drug release from the optimized formulations showed a significantly faster dissolution rate when compared to the market product. In vivo performance of the optimized formulations in rabbits was investigated after filling them into enteric-coated capsules. Ultimately, OFA formulation achieved a 3 times increase in RC oral bioavailability in comparison with the market product, supporting the hypothesis of considering IFNs as promising nanocarriers able to boost the bioavailability of BCS class II drugs.

## 1. Introduction

Rosuvastatin calcium (RC) is an hydroxymethylglutaryl-coenzyme A (HMG CO-A) reductase inhibitor used in the treatment of hyperlipidemia [1]. The oral bioavailability of RC is very low (20%) because of its low aqueous solubility and liability to be extensively metabolized in the liver by means of oxidation, glucuronidation, and lactonization [2]. Regarding stability, RC marketed tablets have a shelf-life of 3 years while it is stable for 30 days in the suspension form [3].

Several approaches have been developed to enhance the solubility of class II drugs in the GIT physiological conditions, including the formulation and development of nanocrystals, solid dispersion on a soluble carrier, and single and mixed micelles. Nanocrystals are prepared either by the bottom-up technique or top-down technology. In the case of bottom-up technique, it is necessary to find two miscible solvents where one of them can solubilize the drug and the other acts as an anti-solvent [4]. This cannot be applied to all active ingredients. On the other hand, top-down technology by a high-pressure homogenizer consumes a lot of energy and requires recycling of the product several times to obtain a small and uniform particle size [5]. Solid dispersions suffer from their tendency to absorb moisture after preparation and this moisture can promote the conversion of the drug from the amorphous or the metastable forms to a stable crystalline form that has low solubility [6]. Single micelles usually have a relatively larger particle size, lower loading capacity, and lower thermodynamic stability, when compared to mixed micelles [7]. Consequently, mixed micelles are more preferred than single micelles but still have some drawbacks related to the use of a relatively large amount of surfactants which may lead to irritation [8]. This can be avoided through the selection of non-ionic surfactants with excellent biocompatibility profiles, e.g., Tween^®^ 80 and cetyl alcohol. Cetyl alcohol is used as a food additive and listed by the FDA as GRAS (generally recognized as safe) [9]. On the other hand, Tween^®^ 80 is safely used as a solubilizer for oil-soluble vitamins and administered with a daily dose of 300 to 500 mg [10].

Tween^®^ 80 is used as a surfactant to enhance the solubility of poorly water-soluble drugs and cetyl alcohol is utilized to impart a lipophilic environment and enhance lymphatic uptake [11]. These nanovesicles (lipotomes) are supposed to be superior over the conventional liposomes due to replacing the main liposomal components, i.e., phosphatidylcholine and cholesterol with Tween^®^ 80 and cetyl alcohol. Phosphatidylcholine liposomes suffer from low stability and high drug leakage in the GIT physiological conditions due to their liability to enzymatic degradation [12,13,14].

A lot of efforts have been exerted to convert micelles and other nanovesicles to solid forms through lyophilization, spray drying, and loading on solid carriers [15,16,17,18]. Getting nanovesicles in a solid stable form requires at least two-steps; preparation of a nanodispersion followed by solidification/drying, which is a complicated and time-consuming process. On the other hand, pro-nanovesicles can be prepared in a single-step which in turn transform into real nanovesicles after administration, e.g., proliposomes and proniosomes [19,20,21].

The aim of this study was to prepare and develop pro-vesicular systems composed of the same components of lipotomes but loaded onto surfaces of a carrier, e.g., mannitol or Aerosil, in an easy and simple single step. The prepared in situ forming nanovesicular (IFN) systems were hypothesized to form nanovesicles in situ after oral administration of enteric-coated capsules containing these pro-vesicles. The coat was used to prevent the drug release in the stomach and release the whole drug dose in the small intestine where the drug is supposed to be directly absorbed through the portal circulation to its site of action in the liver.

## 2. Materials and Methods

### 2.1. Materials

Rosuvastatin calcium was generously supplied by Global Napi Pharmaceutical Company, Cairo, Egypt (Batch number: RV0140614). Cetyl alcohol (CA), Tween 80 (T80), mannitol, Aerosil 200 (Fumed silica), and polyethylene glycol 400 (PEG) were purchased from Sigma–Aldrich, St. Louis, MI, USA. Crestor^®^ tablets, AstraZeneca, Giza, Egypt (Batch number: 170509) were purchased from the Egyptian market. Eudragit L100 was supplied by Rohm GmbH, Darmstadt, Germany.

### 2.2. IFN Preparation

IFN was prepared using a modified thin film hydration technique. In brief, the drug (10 mg), T80 (as a surfactant), and 50 mg CA (as a lipid component) were precisely weighed and dissolved in 10 mL of chloroform:methanol (2:1 *v*/*v*) mixture in a 250 mL round-bottom flask [11]. Then, various amounts of the carrier (mannitol or Aerosil), as shown in Table 1, were directly added into the flask. The organic solvents were gradually evaporated in a rotary evaporator (Rotavapor, Heidolph VV 2000, Burladingen, Germany). The speed was fixed at 150 rpm for 15 min and the temperature was adjusted to 60 °C under vacuum. The organic dispersion was converted into solid within two minutes but stirring under the same conditions continued to ensure the complete volatilization of the organic solvents. Finally, the formed powder was collected and kept in desiccator until inspection.

### 2.3. The Study Statistical Design

The D-optimal statistical design was used to explore the impact of different formulation variables on the characteristics of IFN using Design-Expert^®^ 7 software (Stat-Ease Inc., Minneapolis, MN, USA). Three independent formulation parameters were defined in this design: T80 (X_1_), carrier amounts (X_2_), and carrier type (X_3_). The chosen responses were particle size (Y_1_: PS), zeta potential (Y_2_: ZP), and entrapment efficiency (Y_3_: EE) to ensure that the reconstituted nanovesicles were small in size, physically stable, and capable of entrapping as much drug as possible. This design was composed of 14 formulations with 19 runs as each of the formulations, F1, F2, F9, F10, and F13, was prepared twice as an integral part of the statistical design to adjust the repeatability of the results [22]. Table 1 shows the detailed composition of the prepared IFN formulations. Furthermore, desirability values were determined to simultaneously enhance the traced responses and obtain the composition of the optimized IFN formulations.

### 2.4. Characterization of the Prepared IFN

#### 2.4.1. Measurement of PS, PDI, and ZP

Samples (0.1 g) withdrawn from each RC IFN formulation were hydrated using 10 mL distilled water and then sonicated (Crest Ultrasound, Imola, Italy) for 3 min to convert IFN formulations to aqueous dispersions of nanovesicles. Aerosil powder was removed by filtration before analysis using Whatman filter paper (pore size: 3 μm). PS (Z-average) and PDI were analyzed using the dynamic light scattering technique (Malvern Zetasizer, Worcestershire, UK). Moreover, the ZP of each prepared formulation was measured to indicate their physical stability. Measurements were performed in triplicate at a 90° scattering angle and 25 °C. The analysis was repeated three times, then the average ±SD was recorded.

#### 2.4.2. Measurement of EE

Samples from RC IFN formulations (0.1 g) were reconstituted using 10 mL distilled water and then sonicated for 3 min. The reconstituted RC nanovesicles were separated from the unentrapped drug, the insoluble carrier, in the case of Aerosil IFN formulations, and any possible aggregates by filtering 5 mL of the formed dispersion using a Whatman^®^ membrane filter (pore size: 0.45 μm). Because of its poor water solubility, the unentrapped RC was precipitated and settled on the surface of the filter paper, while RC containing vesicles passed into the filtrate through the filter paper pores [23,24,25,26]. Then, sample aliquots from the filtrate were diluted with methanol and underwent sonication for 3 min to disrupt the nanovesicular structure. RC concentration was spectrophotometrically analyzed in the resulting filtrate at a predetermined λ_max_ of 290.6 nm. RC entrapped percentage was determined using the following equation:

(1) $$\mathrm{EE}\%=\frac{\mathrm{weight}\;\mathrm{of}\;\mathrm{the}\;\mathrm{drug}\;\mathrm{in}\;\mathrm{the}\;\mathrm{IFN}}{\;\mathrm{weight}\;\mathrm{of}\;\mathrm{added}\;\mathrm{drug}\;\mathrm{during}\;\mathrm{preparation}}\;\times \;100.$$

#### 2.4.3. Flowability of the prepared IFN

An exact measured quantity of 5 g of each IFN formulation, which passed through a 1 mm sieve, was settled in a cylindrical measure and the occupied volume was recorded as *V*_i_ (initial volume). Then, the graduated cylinder was tapped manually until a stable volume was attained, and the powder volume was then recorded as a final volume (*V*_f_). Carr’s index (CI) was then calculated as follows [27]:

(2) $$Carr^{\prime }s\;Index=\left[1-\left(\frac{V_{f}}{V_{i}}\right)\right]\times 100$$

The Hausner ratio (HR) was determined using the following equation [28]:

(3) $$HR=\frac{V_{i}}{V_{f}}$$

Both the CI and HR experiments and calculations were repeated three times for all the prepared formulations, thereafter the average values ±SD were recorded.

### 2.5. Characterization of the Optimized IFN Formulations

#### 2.5.1. Imaging 

Surface morphology of the optimized IFN formulations was investigated using SEM (JXA-840; JEOL, Japan), after being coated with gold under vacuum [29]. On the other hand, samples of the IFN formulations were reconstituted with 10 mL water and sonicated for 3 min. The reconstituted samples were placed over copper plates coated with carbon and then stained with 2% *w*/*v* phosphotungistic acid aqueous solution. The stained samples were imaged using a transmission electron microscope (JEOL, Tokyo, Japan) working at a 100 kV accelerating voltage.

#### 2.5.2. In Vitro RC Release from the Optimized IFN Formulations

In vitro RC release from the optimized IFN formulations (equivalent to 5 mg RC), packed in hard gelatin capsules (size 2), was characterized in a USP dissolution apparatus II (Pharm Test, Hainburg, Germany). The dissolution medium was composed of 250 mL hydrochloric acid (pH 1.2). During the dissolution study, the speed of the shaft rotation was fixed at 50 rpm and the temperature was kept at 37 ± 1 °C. The release of RC from capsules loaded with an equivalent amount of RC powder was investigated under the same conditions and considered as a reference [30,31]. Samples, 3 mL each, were withdrawn at different time intervals (5, 10, 15, 20, 30, 45, 60, 90, and 120 min), followed by the addition of an equal volume of the dissolution medium. The concentrations of RC in the collected samples were spectrophotometrically analyzed at a λ_max_ of 290.6 nm. The drug release profiles from the optimized formulations and the drug powder were compared using the similarity factor (*f*_2_) [32].

### 2.6. Preparation of Enteric-Coated Capsules Filled with the Optimized RC IFN

The optimized IFN formulations, either containing mannitol (OFM) or Aerosil (OFA), were filled into hard gelatin capsules with quantities equivalent to 5 mg RC. The coating process of the capsules was started by sealing the body and the cap using a brush dipped in 5% ethanolic solution of ethyl cellulose. Thereafter, capsules were spray-coated in a pan (Royal Artist, Mumbai, India) rotating at 30 rpm [33]. The coating solution consisted of 12.5% *w*/*v* ethanolic solution of Eudragit L100 plasticized with 2.5 % *v*/*v* PEG 400. It was atomized under compressed air from a painting pistol (APT, H.D power tools, Zhejiang, China) with a rate of 4 mL/min. Throughout the spraying process, the organic solvent was continuously evaporated under a stream of hot air. The endpoint was to achieve a 40% weight increase of the sprayed capsules.

### 2.7. Characterization of the Prepared Enteric-Coated Capsules

#### 2.7.1. Weight Variation and Content Uniformity

Coated capsules (from each optimized IFN formulation) were weighed separately and then the mean weight was determined to detect the degree of weight variation [34].

The content uniformity test was carried out according to BP [34]. The content of 10 capsules (pre-filled with each optimized formulation) was individually dissolved in a volumetric flask containing 100 mL methanol under vortexing (VM-300, Gemmy Industrial Corp., Taiwan) for 3 min. Samples of the resulting liquids were filtered, then samples of the filtrate were diluted with a suitable volume of methanol [35,36]. RC content was spectrophotometrically estimated by determining its UV absorbance at λ_max_ 290.6 nm. The average drug content ±SD was calculated according to this equation [37]:

(4) $$\mathrm{Content}\;\mathrm{uniformity}\;=\frac{\mathrm{Actual}\;\mathrm{drug}\;\mathrm{amount}\;\mathrm{in}\;\mathrm{capsules}\;}{\mathrm{Theoretical}\;\mathrm{drug}\;\mathrm{amount}\;\mathrm{in}\;\mathrm{capsules}\;}\times \;100$$

The drug content should range from 85% to 115% of the labeled potency.

#### 2.7.2. In Vitro RC Release from the Enteric-Coated Capsules

The integrity of the applied coat was investigated through the conduction of an in vitro release study for the enteric-coated capsules in 0.1 N HCl for 2 h. Moreover, RC release in citrate buffer (pH 6.6) was investigated under the same conditions applied to the uncoated capsules [38,39]. Crestor^®^ Tablets and capsules loaded with drug powder were considered as references for the drug release rate and extent. Samples were withdrawn at different time intervals of 5, 10, 15, 20, 45, 60, 90, 120, 125, 130, 135, 140, 150, 180, and 240 min. RC concentrations in the collected samples were spectrophotometrically analyzed at λ_max_ = 290.6 nm. The release study was performed in triplicates and the mean percentages dissolved (±SD) were presented versus time.

### 2.8. Bioavailability Study of RC

The study included three groups of healthy New Zealand male rabbits (3–4 kg), three rabbits in each group. Groups I and II orally received enteric-coated capsules filled with the OFM and OFA formulations, respectively, while group III administered the market product (Crestor^®^, AstraZeneca, Giza, Egypt). The equivalent dose given to the rabbits was 0.2 mg/kg and it was calculated based on the body surface area [40].

Rabbits were fasted overnight before receiving the capsules, with free access to water. The study procedure was ethically approved by the ethics committee of the Faculty of Pharmacy, Cairo University (Acceptance No. PI 1783, 15 July 2016). After oral administration of capsules, blood samples were withdrawn from the rabbits’ ear veins to pre-heparinized glass tubes at different time intervals. The collected blood samples were centrifuged at a speed of 4000 rpm for a period of 10 min at a temperature of 4 °C. Then, plasma was transmitted to 5 mL plastic tubes and frozen at −70 °C until the analysis time. A cross-over design was applied with a wash-out period of a week.

Atorvastatin (50 µL–100 ng/mL) was added to the plasma samples as an internal standard [41]. RC and atorvastatin were extracted using ethyl acetate (4 mL). LC-MS/MS (API-4000, AB Sciex, Foster, CA, USA) was utilized to analyze the drug and the method has been validated in terms of the linearity, accuracy, and lower limit of quantification (LLOQ) [42]. The mobile phase was composed of acetonitrile and 0.1% formic acid in water, in ratio of 4:1 *v*/*v*. The column was Zobrax Eclipse Plus with dimensions of 4.6 × 50 mm and a particle size of 5 µm (Agilent, CA, USA). The injection volume was 15 μL and the flow was isocratic with a rate of 0.9 mL/min.

A non-compartmental pharmacokinetic model was utilized to analyze the pharmacokinetic parameters of RC after oral administration of the capsules containing the optimized formulations and the market product using Kinetica^®^ software version 5 (Thermo Fisher Scientific Inc., Waltham, MA, USA) [43].

## 3. Results and Discussion

### 3.1. Characterization of the Prepared IFN

#### 3.1.1. Measurement of PS, PDI, and ZP

Mean PS of all formulations was in the nano range between 41.0 and 364.9 nm as illustrated in Table 1. PS values were fitted to the polynomial analysis utilizing a quadratic model. Adequate precision was 29.9. The difference between the values of the predicted R^2^ (0.9012) and the adjusted R^2^ (0.9617) was reasonable, indicating a good correlation between the dependent and the independent variables [24]. PS analysis was correlated to the studied formulation factors according to the following equation:

PS = 352.0 − 68.4X_1_ + 25.7X_2_ + 3.6X_3_ + 41.0X_1_.X_2_ + 13.2X_1_.X_3_ + 0.1X_2_.X_3_ + 65.1X_1_^2^ − 216.2X_2_^2^ (5)

**Figure 1:**
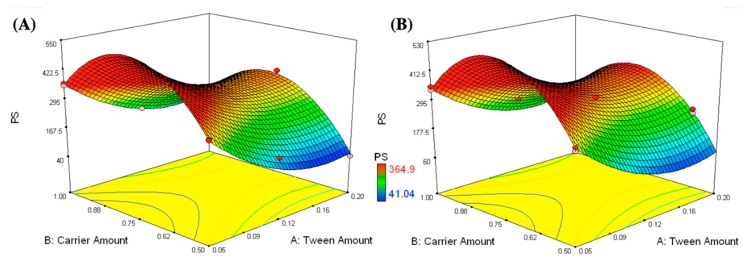
Response surface plots for the effects of T80 (X_1_) and carrier amounts (X_2_) on the mean particle size of the IFN formulations loaded on different types of carriers (X_3_); mannitol (**A**) and Aerosil (**B**).

The independent variables were the amounts of T80 (X_1_) and carrier (X_2_) and showed significant effects on the mean particle size (*p*-value < 0.0001), as shown in Figure 1. It was obvious that the mean PS was significantly decreased by increasing the T80 amount and decreasing the carrier amount. The lowest particle size values were observed in formulations containing the highest amount of T80 and the least amount of carrier, such as formulation F6 (0.2 g T80 and 0.5 g mannitol) and formulation F13 (0.2 g T80 and 0.58 g Aerosil). Increasing Tween^®^ 80 might lead to an increase in the surface activity, forming nanovesicles with smaller PS. Similar results were observed by Negi et al. while investigating the impact of T80 concentration on the vesicle size of venlafaxine niosomes [44]. Rahman et al. also stated that vesicle size decreased with increasing the surfactant concentration [45]. On the other hand, the observed increase in the PS by increasing the carrier amount was statistically significant. For mannitol, the significant size increase might be due to its tendency to crystallize in a concentration higher than 0.4 M (0.7 g in 10 mL) and this could illustrate the increase in the PS of the formulations containing 0.81 and 1 g mannitol after reconstitution in 10 mL distilled water [46]. Regarding Aerosil, the increase in the PS can be explained in terms of the affinity of Aerosil to agglomerate at high concentration and this is in harmony with the results reported by Varshosaz et al. [47].

Moreover, PDI values of all formulations were determined and are illustrated in Table 1. PDI values were within the range of 0.127 to 0.325 which reflected an acceptable particle size uniformity [48]. Statistical analysis of the obtained data showed that the studied independent variables had no significant impact on the PDI values.

**Figure 2:**
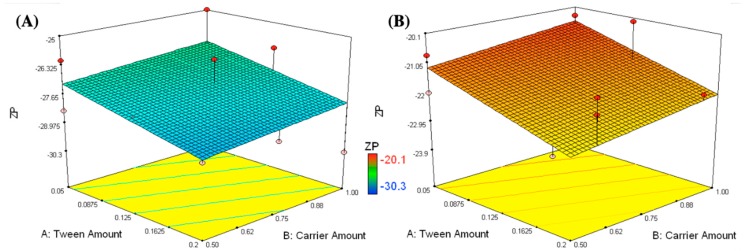
Response surface plots for the effects of T80 (X_1_) and carrier amounts (X_2_) on the zeta potential of formulations loaded on different types of carriers (X_3_); mannitol (**A**) and Aerosil (**B**).

ZP values of the prepared formulations (pH 6.1–6.8) ranged between –30.3 and –20.1 mV, as shown in Table 1 and Figure 2. The higher the zeta potential values, the higher the repulsion forces, which prevent aggregation of the nanovesicles [49,50]. A linear model was applied to the ZP findings. The following equation describes the effects of the different studied formulation variables on ZP:

ZP = −24.6 − 0.7X_1_ + 0.3X_2_ + 3.0X_3_ (6)

According to the factorial ANOVA analysis, the carrier type (X_3_) was the only factor having a significant effect on the ZP of the prepared nanovesicles (*p*-value < 0.0001). Mannitol containing formulations have significantly higher ZP than those containing Aerosil. This might be due to the covering of the reconstituted nanovesicles by a dense layer of negatively charged hydroxyl groups belonging to mannitol. This was in harmony with the previous findings reported by Gawali et al., who studied the effect of mannitol on the stabilization of iron nanoparticles [51]. On the other hand, formulations loaded on Aerosil had weak negative charges despite silanol groups being present in its structure. This might be due to the removal of Aerosil by filtration before measurement of the ZP values.

#### 3.1.2. Measurement of EE

**Figure 3:**
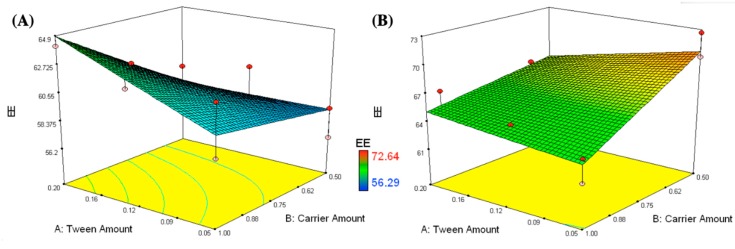
Response surface plots for the effects of T80 (X_1_) and carrier amounts (X_2_) on the entrapment efficiency of the formulations loaded on different types of carriers (X_3_); mannitol (**A**) and Aerosil (**B**).

EE percentages of all RC prepared formulations were within the range of 56.3% to 72.6%, as displayed in Table 1 and Figure 3. A two-factor interaction was utilized to analyze the obtained EE values. Design-Expert software was utilized to estimate the adequate precision (10.6), whereas a value >4 is desired [52]. The predicted R^2^ (0.6274) matched with the adjusted R^2^ (0.8002), indicating the competence of the model to predict response values [53]. The EE findings were analyzed utilizing the following equation:

EE% = 63.6 + 0.3X_1_ − 0.2X_2_ + 3.1X_3_ + 1.2X_1_.X_2_ − 0.8X_1_.X_3_ − 2.2 X_2_.X_3_ (7)

Changing the carrier type (X_3_) had a statistically significant effect on the entrapment efficiency with a *p*-value < 0.0001. Mannitol containing formulations had lower EE values than Aerosil containing ones. This might be due to the possible RC salting out by the high mannitol concentrations incorporated into the IFN formulations [54]. Similar results were observed by Huang et al. while investigating the effect of mannitol on the encapsulation efficiency of Calcein in liposomes [46].

#### 3.1.3. Flowability of the Prepared IFN

CI is commonly used to predict the flowability of powders. All the prepared IFN formulations exhibited excellent to pass flowability according to the pharmacopoeial ranges of both CI (<26%) and HR (<1.35), as shown in Table 1 [55,56,57]. This might be due to the excellent flowability of the utilized carriers; mannitol and Aerosil represent the main bulk of the formulations’ weight [58]. The high flowability of the prepared IFN facilitated the recovery of more than 95% of the utilized excipients after preparation.

### 3.2. Selection of the Optimized IFN Formulations

**Figure 4:**
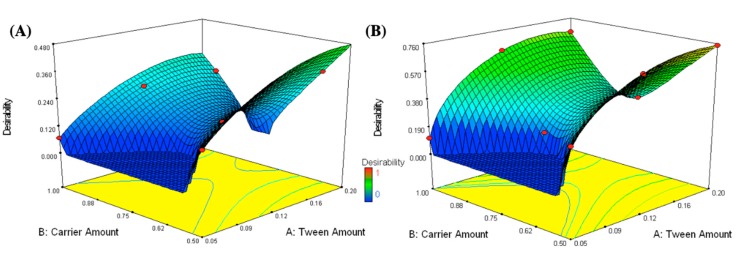
Response surface plots for the effects of T80 (X_1_) and carrier amounts (X_2_) on the desirability of the formulations loaded on different types of carriers (X_3_); mannitol (**A**) and Aerosil (**B**).

**Figure 5:**
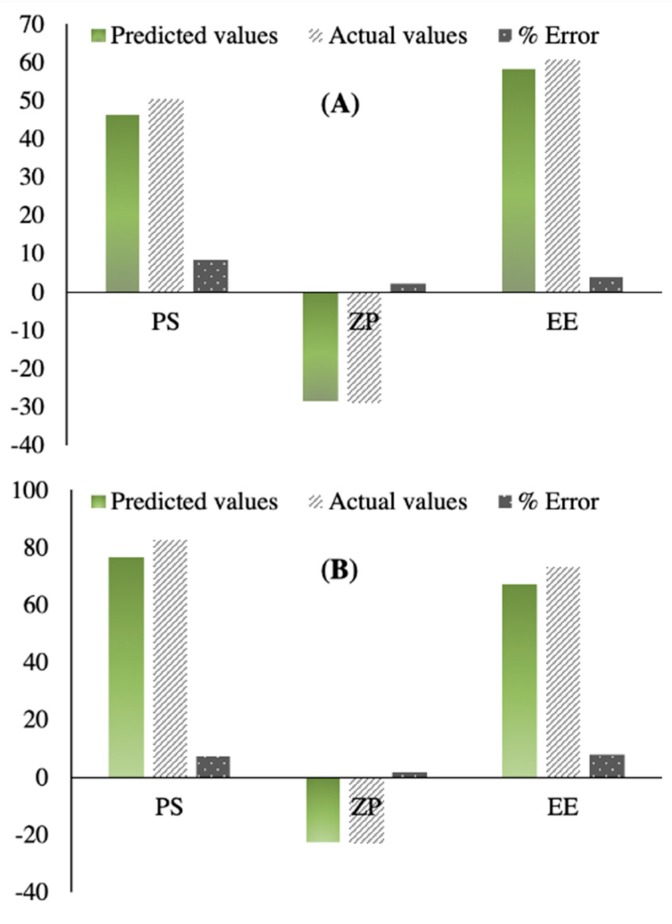
The correlation between the predicted and the actual values of the particle size, zeta potential, and entrapment efficiency of OFM (**A**) and OFA (**B**).

It is a challenge to simultaneously optimize all independent variables because the optimum conditions optimizing a response could adversely affect the other [59]. The desirability was determined to choose the optimized formulations that collectively have maximal EE and ZP and minimal PS values. The maximum desirability values were 0.754 and 0.478 for the optimized formulations containing 0.05 g CA, 0.18 g T80, in addition to 0.5 g mannitol (OFM) or Aerosil (OFA), respectively, as demonstrated in Figure 4. The optimized formulations were prepared and subjected to further in vitro and in vivo characterizations. PS, ZP, and EE were measured for the prepared optimized formulations (OFM and OFA) and the results were compared to the predicted values generated from Design-Expert software, as shown in Figure 5A,B, respectively. The percentages of error were less than 10% in each of the measured responses, indicating the design’s capability to navigate formulation factors and predict relatively accurate outputs.

### 3.3. Characterization of the Optimized IFN Formulations

#### 3.3.1. Imaging

**Figure 6:**
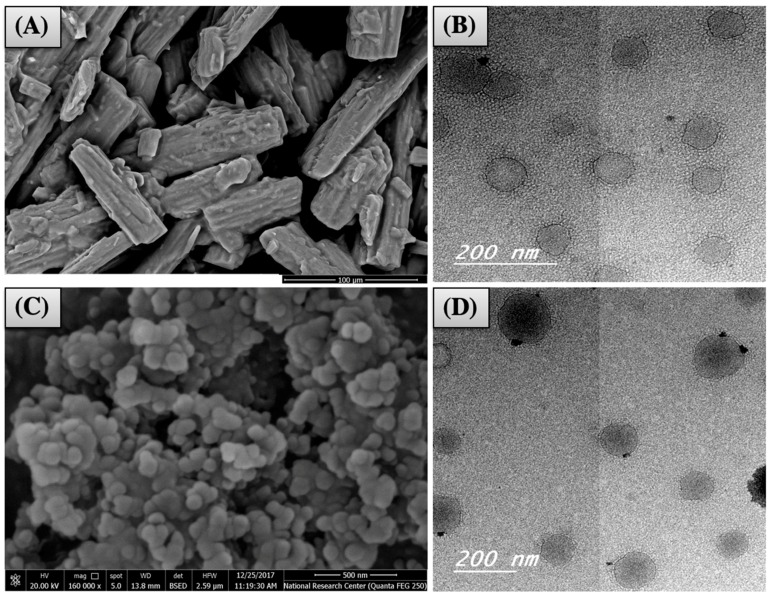
Imaging of OFM (**A**: by SEM and **B**: by TEM) and OFA (**C**: by SEM and **D**: by TEM).

SEM was utilized to examine the morphology of the optimized IFN formulations. SEM of OFM had aggregates of typical rods or elongated crystals of mannitol (Figure 6A). SEM of OFA showed spherical Silanol agglomerates, as illustrated in Figure 6C. On the other hand, the reconstituted nanovesicles appeared to be spherical with a slightly irregular surface and homogenous PS under the transmission electron microscope, as shown in Figure 6B,D. The PS values (50–100 nm) were in harmony with those measured by Zetasizer. No aggregates were observed, and this could be due to the relatively high ZP observed in the optimized formulations (OFM and OFA).

#### 3.3.2. In Vitro RC Release from the Optimized IFN Formulations

**Figure 7:**
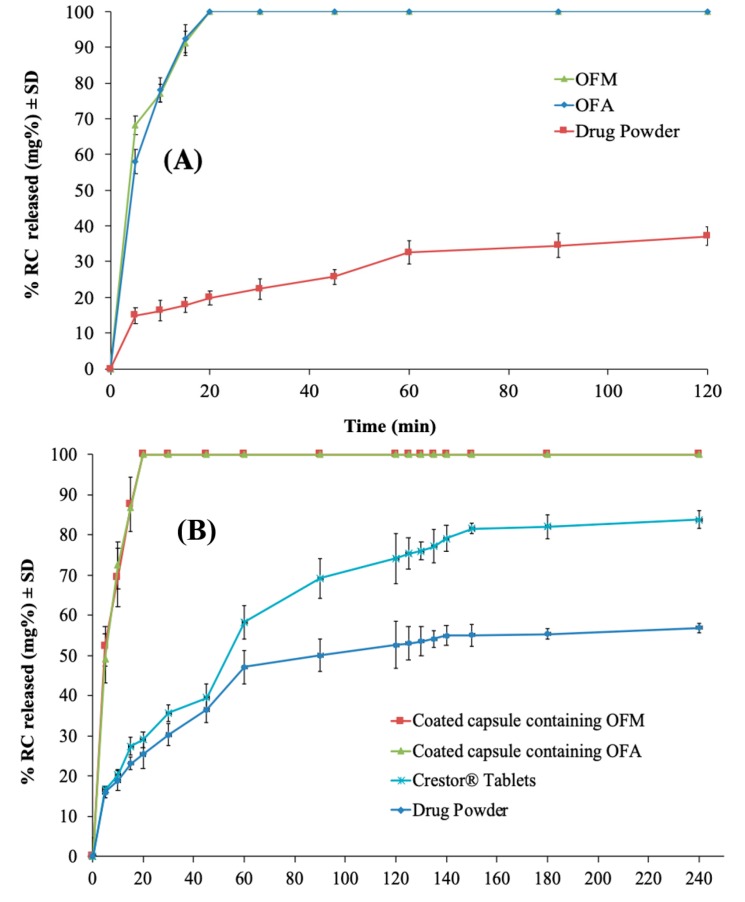
Release profile of RC from the optimized IFN formulations in 0.1N HCl (**A**), in comparison with the drug powder, and from the enteric-coated capsules containing the optimized formulations in citrate buffer (pH 6.6) (**B**), in comparison with the market product (Crestor^®^ Tablets) and the drug powder.

Figure 7A demonstrates the RC release from the optimized IFN systems (OFM and OFA), in comparison with the drug powder in 0.1N HCl (pH 1.2). The selected medium did not provide sink conditions for the drug dissolution to demonstrate the formulation effect of enhancing the drug dissolution rate [60,61,62]. Dissolution was not compared to the RC market product as 0.1 N HCl is not an official medium for drug dissolution [63]. The release profiles of RC from OFM and OFA are superimposing, indicating that the carrier type and either being soluble or not had no significant effect on the drug release from the reconstituted nanovesicles. Similar results were reported by Bremmell et al., who observed the ability of mannitol and silica to retain the drug release from silica nanoparticle–lipid–mannitol hybrid microparticles [58]. The OFM formula had a zero order release (R^2^ = 0.9907) while OFA followed Higuchi diffusion (R^2^ = 0.9901). On the other hand, the drug suspension showed a first order drug release (R^2^ = 0.9800). Conforming to the FDA, both formulations released 85% or more of the drug within the first 15 min of the release study, and the profiles are considered similar without calculation of *f*_2_ [32]. Where all the drug (100%) had been released from both OFM and OFA after just 20 min, only 19.85% of the drug was released from the capsules loaded with RC powder during the same period. Additionally, the drug powder released nearly 37.15% of the labeled dose during the entire release period (120 min), with *f*_2_ values of 10 and 11 with the OFM and OFA, respectively. The low drug release from the drug powder might be due to the low drug solubility in the employed acidic medium [64]. Consequently, the obtained findings indicated that the optimized IFN formulation significantly improved the rate and extent of RC release when compared to its powder.

### 3.4. Characterization of the Prepared Enteric-Coated Capsules

#### 3.4.1. Weight Variation and Content Uniformity

According to the BP, the examined capsules can pass the test if just two capsules or less deviate from the average weight by >10%, and none deviates by >20% [34]. None of the investigated capsules (filled with optimized formulations) deviated from the average weight by >10%. This revealed that all formulations complied with the pharmacopoeial limits.

A content uniformity test was performed according to the BP and RC content of all examined capsules found to be within the range of 85% to 115%, complying with the pharmacopoeial limits [34].

#### 3.4.2. In Vitro RC Release from the Enteric-Coated Capsules

Capsules were enteric-coated to release the whole drug dose only in the small intestine where the drug is supposed to be directly absorbed through the portal circulation to the liver (the site of action) [65]. The enteric-coated capsules showed zero release in 0.1 N HCl, indicating the capability of the applied enteric coat to prevent RC release in the stomach. This could be attributed to the pH-dependent solubility of Eudragit L100, which was used as an enteric coat, where it dissolves at pH ≥ 6 [11,66]. On the other hand, Figure 7B reveals the release profiles of RC from the coated capsules containing OFM and OFA formulations, compared to the market product and the drug powder, in citrate buffer (pH 6.6). A significant increase in the drug release rate and extent was observed with a similarity factor of 14 between each of the optimized formulations and the market product. The optimized formulations released all the loaded drug within 20 min while only 29.05% was released from the market product and 25.44% from the capsules loaded with the drug powder. This could indicate the significant enhancement achieved in the drug rate and extent regardless of the surrounding physiological pH although the RC originally has a pH-dependent solubility [64].

### 3.5. Bioavailability of RC

**Figure 8:**
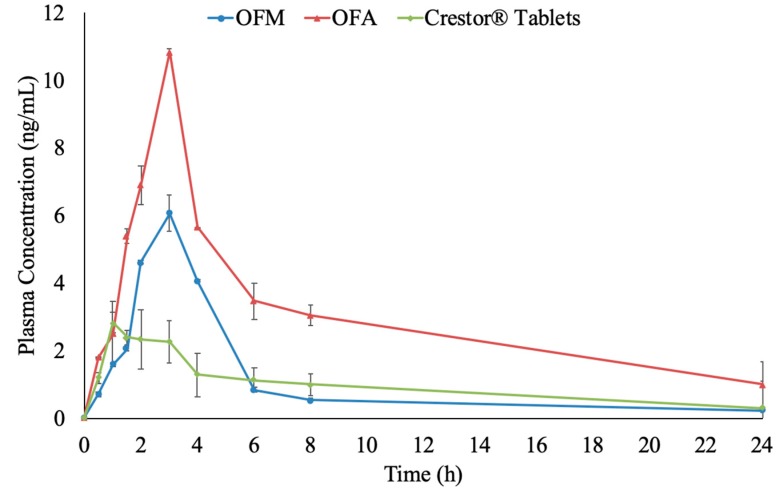
Plasma level-time curve of RC after a single oral dose of enteric-coated capsules filled with both optimized (OFM and OFA) formulations and the market product (Crestor ^®^ Tablets).

The utilized LC-MS/MS analysis method had a correlation coefficient of 0.9983 and an LLOQ of 0.1 ng/mL. The calibration curve covered a concentration range up to 100 ng/mL. Accuracy was in the range of 100% ± 15 for the selected quality control samples (QCL: 0.3 ng/mL, QCM: 50 ng/mL, QCH: 80 ng/mL). The pharmacokinetic parameters of RC following oral administration of coated capsules containing OFM and OFA formulations and the market product (Crestor^®^) were investigated. The results showed that the formulated capsules showed superior bioavailability when compared to the market product in terms of C_max_ and AUC_0–∞_. C_max_ values of OFM (6.08 ± 0.52 ng/mL) and OFA (10.84 ± 1.02 ng/mL) formulations were significantly higher than the market product (2.83 ± 0.94 ng/mL), as demonstrated in Table 2 and Figure 8 (*p*-values < 0.001). The significantly high t_max_ of the optimized formulations (3 h), compared to the market product (1 h), indicated that the optimized formulations delivered RC at a significantly slower rate than the market product (*p*-value < 0.001). This was typically matching with the enteric coat applied to the optimized formulations, which delayed the drug release and consequently, the in vivo absorption by 2 h. There was no significant difference in the elimination t_1/2_ and K_e_ between the optimized formulations and the market product (*p*-values > 0.05).

The AUC_0–∞_ of the optimized formula containing Aerosil (OFA) showed a three-fold increase over that of the market product with a *p*-value < 0.001. This might be due to the incorporation of both T80 and CA. T80 increased the solubility of RC and as a result increased its dissolution and absorption rate and extent. On the other hand, CA as a lipid moiety, could facilitate intestinal absorption through lymphatic uptake as previously observed and reported by Garg et al., Makwana et al. and Baek et al. [67,68,69,70]. Moreover, Siram et al. stated that nanovesicles and nanoparticles with a PS less than 100 nm could be capable of achieving better lymph uptake, which applies to the nanovesicles reconstituted from the optimized IFN formulations [71]. Enhancing the lymphatic uptake of RC could bypass the first pass effect and hence increase the oral bioavailability [72].

On the other hand, the mean AUC_0–∞_ in the case of the optimized formula containing mannitol (OFM) was not significantly different from the market product with a *p*-value of 0.230. This insignificant increase in the AUC values could be attributed to the possible reduction in the GIT transit time in the presence of mannitol which could counteract the increase in the drug dissolution rate [73,74]. It was previously reported that mannitol in small concentrations could reduce the bioavailability of drugs, especially those absorbed from the small intestine [75,76].

## 4. Conclusions

IFN is a stable and easily prepared platform able to form hybrid nanovesicles after oral administration. The formed nanovesicles are mainly composed of lipophilic fatty alcohol and a hydrophilic surfactant, providing a tailored microenvironment for the entrapment of a wide range of active ingredients. Significant improvements in the drug dissolution rate were observed in the optimized IFN formulations when compared to the market RC product. Drug bioavailability was increased three times in the case of the IFN optimized formula loaded on Aerosil. Hence, IFN can be considered as a promising nanocarrier to enhance the oral bioavailability of BCS class II drugs suffering from a low solubility in physiological media.

## Figures and Tables

**Table 1:** Experimental runs, independent variables, and measured responses of the D-optimal experimental design for the IFN formulations.

Formulations	X_1_: Tween 80 Amount (g)	X_2_: Carrier Amount (g)	X_3_: Carrier Type	Y_1_: PS (nm)	Y_2_: ZP (mV)	PDI	Y_3_: EE (%*w*/*w*)	CI (%)	HR
F1	0.05	0.5	Mannitol	304.7 ± 11.6	−28.4 ± 1.1	0.325 ± 0.05	56.3 ± 3.5	12.5 ± 0.2	1.1 ± 0.02
				299.4 ± 10.4	−26.1 ± 1.0	0.299 ± 0.03	58.7 ± 4.2	12.4 ± 0.1	1.2 ± 0.01
F2	0.05	1		364.9 ± 9.1	−25.0 ± 0.5	0.275 ± 0.03	58.5 ± 4.1	18.7 ± 0.3	1.2 ± 0.02
				354.8 ± 6.5	−27.0 ± 0.0	0.127 ± 0.05	62.9 ± 5.5	16.7 ± 1.0	1.2 ± 0.04
F3	0.12	0.81		333.6 ± 13.9	−26.2 ± 1.0	0.241 ± 0.02	60.9 ± 1.8	20.0 ± 0.4	1.2 ± 0.03
F4	0.12	0.52		124.6 ± 1.0	−27.9 ± 0.8	0.236 ± 0.03	60.8 ± 5.7	10.0 ± 0.3	1.1 ± 0.02
F5	0.12	1		171.3 ± 14.5	−26.2 ± 1.1	0.127 ± 0.05	63.4 ± 3.3	17.6 ± 0.6	1.2 ± 0.01
F6	0.2	0.5		41.0 ± 0.9	−28.7 ± 0.6	0.221 ± 0.04	59.1 ± 2.7	20.0 ± 0.4	1.2 ± 0.04
F7	0.2	0.75		348.2 ± 14.5	−28.8 ± 1.1	0.316 ± 0.05	64.4 ± 3.3	17.6 ± 0.6	1.2 ± 0.01
F8	0.2	1		180.5 ± 0.9	−30.3 ± 0.6	0.174 ± 0.04	72.1 ± 2.7	20.0 ± 0.4	1.2 ± 0.04
F9	0.05	0.5	Aerosil	278.3 ± 10.6	−20.8 ± 0.1	0.271 ± 0.01	70.0 ± 3.5	18.9 ± 0.1	1.2 ± 0.01
				264.7 ± 12.4	−22.1 ± 0.2	0.249 ± 0.01	72.6 ± 1.6	17.6 ± 0.1	1.1 ± 0.02
F10	0.05	1		337.9 ± 2.7	−21.8 ± 0.7	0.197 ± 0.04	61.7 ± 1.2	10.2 ± 0.3	1.2 ± 0.02
				351.2 ± 3.5	−20.4 ± 0.8	0.267 ± 0.03	64.2 ± 0.8	11.6 ± 0.3	1.2 ± 0.01
F11	0.12	0.96		240.0 ± 6.9	−20.1 ± 0.5	0.313 ± 0.02	65.2 ± 2.1	13.8 ± 0.5	1.2 ± 0.04
F12	0.13	0.68		329.8 ± 9.5	−23.9 ± 0.1	0.304 ± 0.05	66.8 ± 6.3	5.4 ± 0.3	1.0 ± 0.03
F13	0.2	0.58		235.5 ± 11.7	−21.1 ± 1.0	0.245 ± 0.01	65.2 ± 3.9	3.3 ± 0.7	1.0 ± 0.05
				219.4 ± 7.0	−21.6 ± 1.1	0.249 ± 0.01	67.2 ± 7.0	3.5 ± 0.7	1.1 ± 0.04
F14	0.2	0.95		266.0 ± 5.7	−21.9 ± 0.4	0.233 ± 0.06	66.8 ± 1.1	10.2 ± 0.1	1.1 ± 0.01

All formulae containing 0.01 g drug and 0.05 g cetyl alcohol; The independent variables were the amount of T80 (X_1_), amount (X_2_), and type of the carrier (X_3_). While the selected dependent variables were the particle size (Y_1_), Zeta potential (Y_2_), and entrapment efficiency (Y_3_).

**Table 2:** RC pharmacokinetic parameters after the oral administration of the coated capsules filled with the optimized formulations containing mannitol (OFM) or Aerosil (OFA), compared to the market product.

Pharmacokinetics Parameters	Treatment (Mean ± SD) ^a^		
	OFM	OFA	The Market Product
*C*_max_ (ng/mL)	6.08 ± 0.52	10.84 ± 1.02	2.83 ± 0.94
AUC_0-24_ (ng.h/mL)	26.22 ± 1.87	71.71 ± 3.98	23.02 ± 1.36
AUC_0-∞_ (ng.h/mL)	29.92 ± 1.24	86.16 ± 5.21	27.38 ± 2.09
*T*_max_ (h)	3.00	3.00	1.00
*K*_e_ (l/h)	0.06 ± 0.00	0.07 ± 0.00	0.07 ± 0.00
*t*_½e_ (h)	10.92 ± 1.13	10.01 ± 1.89	9.63 ± 0.82
MRT	9.80	12.66	12.17
% Relative Bioavailability	109.28%	314.68%	-

^a^ Median of Tmax and MRT are displayed instead of the mean values.
